# Construction and validation of a risk prediction model for metabolic syndrome: a cross-sectional study based on randomized sampling

**DOI:** 10.3389/fendo.2025.1761342

**Published:** 2026-01-16

**Authors:** Jiannan Zhao, Xinhua An, Ling Liu, Jia Meng, Liyong Liu, Yongliang Mu

**Affiliations:** 1 Shijingshan District Center for Disease Control and Prevention, Beijing, China; 2 Wulituo Hospital of Shijingshan District, Beijing, China; 3 Department of Vascular Surgery, Tianjin Union Medical Center, The First Affiliated Hospital of Nankai University, Tianjin, China

**Keywords:** age, male, marry, metabolic syndrome, obese, occupation, risk prediction model

## Abstract

**Objective:**

The prevalence of metabolic syndrome is high among Chinese residents, and it is crucial to understand the current situation and intervene promptly. In this study, we investigated the current status of metabolic syndrome in some regions of China, analyzed related risk factors, and developed a risk prediction model to guide preventive measures.

**Methods:**

A multistage stratified cluster random sampling method was used to select 3541 permanent residents aged 18-79 years from a district in Beijing for face-to-face questionnaire surveys, physical examinations, and laboratory tests. All participants were randomly divided into training and validation sets. Correlation analysis and multivariate logistic regression were employed to identify risk factors for metabolic syndrome, and a column-line graph prediction model was developed. The discriminative ability and predictive accuracy of the model were assessed by receiver operating characteristic (ROC) curve and calibration curves.

**Results:**

The prevalence of metabolic syndrome in this study was 18.4%. The results of multivariate logistic regression analysis showed that increasing age, being male (OR = 1.827), being overweight (OR = 4.865), being obese (OR = 11.482), hazardous alcohol consumption (OR = 1.673), marital/cohabitation history, and specific occupations (agriculture, forestry, fisheries, and water production, and unemployed) were independent risk factors for metabolic syndrome (*P* < 0.05). The column-line graph prediction model, constructed accordingly, performed well, and the model indicated that BMI and age were the most significant risk factors for metabolic syndrome. The results of model validation showed that the AUCs of the training and validation sets were 0.815 (95% CI: 0.795-0.836) and 0.787 (95% CI: 0.756-0.818), respectively, indicating that the model performed well in discriminating. The model exhibited a good fit on the training set (with 1000 resamples via the Bootstrap method; calibration slope = 1.002, calibration intercept = 0.005; Hosmer-Lemeshow test, *P* = 0.127 > 0.05). The initial validation set showed signs of slight overfitting, and after probability calibration using the Platt Scaling method, the model’s calibration performance was significantly improved (calibration slope = 0.92, calibration intercept = -0.03; Hosmer-Lemeshow test, *P* = 0.082 > 0.05). The Brier scores of the training set and validation set were 0.120 and 0.136, respectively, with mean absolute errors (MAE) of 0.240 and 0.256, indicating that the model had favorable predictive accuracy and stability.

**Conclusions:**

The metabolic syndrome column-line diagram risk prediction model constructed in this study, based on multivariate logistic regression analysis, has good discriminative ability and high prediction accuracy. The model shows that a large proportion of the current risk factors for metabolic syndrome are modifiable, and that the risk of metabolic syndrome in high-risk groups, such as the elderly, men, people with marital/cohabitation histories, and people with specific occupations, can be reduced through behavioral and lifestyle interventions. This model can provide a scientific basis for the early identification of high-risk groups for metabolic syndrome and has an important guiding value for targeted preventive interventions.

## 1 Introduction

Metabolic syndrome(MetS) is a group of clinical syndromes characterized by multiple risk factors such as obesity, diabetes/insulin resistance, hypertension, and dyslipidemia (1). These metabolic factors interact with each other and lead to serious health problems in the body, significantly increasing the risk of cardiovascular disease and type II diabetes (2). It has been estimated that patients with metabolic syndrome have a 2-fold higher risk of death than those without metabolic syndrome, and their risk of heart disease or stroke is three times higher (3). In addition, the risk of type II diabetes mellitus in patients with metabolic syndrome is five times higher than that of normal people (4).WHO points out that chronic non-communicable diseases are responsible for a total of 74% of deaths globally, of which cardiovascular diseases are the leading cause of death (5). Hence, the prevention and control of metabolic syndrome are crucial for reducing the incidence of disease in humans and lowering mortality. Epidemiologic surveys have shown that the prevalence of metabolic syndrome in people aged 20 years and older in China is 31.1% (6). However, the prevalence varies in different regions due to the influence of different demographic characteristics and lifestyles. In this paper, a population-based study was conducted to understand the current prevalence of metabolic syndrome in a district of Beijing and to analyze its risk factors further and construct a risk prediction model. The aim was to obtain persuasive public health information and provide targeted preventive and control measures.

## 2 Objects and methods

### 2.1 Survey objects and sampling design

This study was carried out in a district of Beijing in 2024. The survey object was permanent residents aged 18-79 years old in the district, with a sample size formula of N=(U^2^ p(1-p))/d^2^ deff, taking U = 1.96, p=10.6% (according to the results of the 2017 Beijing Adult Chronic Disease and its Risk Factor Monitoring, the Beijing prevalence of adult diabetes is 10.6%), d=0.02, deff=1.5, considering gender stratification, the response rate was set to 80%, and the sample size was calculated to be 3413.

A multi-stage stratified whole cluster random sampling design was employed. In the first stage, PPS sampling proportional to population size was used to randomly select three communities from each of the nine streets in our district. In the second stage, each community was divided into some residential groups (with at least 127 households in each group), and one residential group was selected from each community by simple randomization. In the third stage, one resident was selected from each household by the KISH table method. A total of 3,635 subjects were enrolled in the actual survey, 95 samples were excluded from the final analysis due to missing values in at least one variable, and listwise deletion was adopted to address the missing data. Consequently, 3,541 samples were ultimately enrolled in the study analysis, with an overall missing rate of 2.61%. Given that this missing rate was far below the critical threshold recommended by methodological guidelines (5%–10%) and that the results of Little’s MCAR test indicated that the missing data mechanism was missing completely at random (MCAR, *P* = 0.76), the exclusion of these samples would not introduce any bias or impact the validity of the study results.

This study was approved by the Ethics Review Board of the BJCDC (No. 5 of 2017). This project is supported by the Beijing Municipal Finance Program.

### 2.2 Survey content and methods

The survey includes a questionnaire survey, physical examination, and laboratory testing. The questionnaire survey is conducted by a uniformly trained and qualified investigator who inquires face-to-face about the basic situation of the respondents, including basic personal information, behavioral risk factors (smoking, alcohol consumption, dietary intake, sleep), and the prevalence of major chronic diseases (hypertension, diabetes mellitus, dyslipidemia), etc. The physical examination was conducted by the investigator using a uniform standard to measure the height, weight, waist circumference, and blood pressure of the respondents, with body mass index BMI = weight (kg)/height^2^ (m^2^). Waist circumference was measured at the horizontal position of the mid-axillary line between the lower edge of the rib arch and the midpoint of the iliac crest line. Blood pressure was measured according to the methods recommended in the Chinese Guidelines for Blood Pressure Measurement, and the average of the three measurements was taken as the final blood pressure value. Laboratory tests required the collection of fasting venous blood from the investigated subjects. Blood samples were collected, centrifuged, and split, and tested for fasting blood glucose, total cholesterol, LDL cholesterol, HDL cholesterol, triglycerides and so on.

### 2.3 Diagnostic criteria for metabolic syndrome

Considering the differences in the criteria for determining obesity in different populations and races, this paper uses the diagnostic criteria of the Diabetes Branch of the Chinese Medical Association (7), which can be diagnosed as metabolic syndrome with the following three or more items: (1) Abdominal obesity (i.e., central obesity): waist circumference ≥90 cm for men and ≥85 cm for women; (2) Hyperglycemia: fasting blood glucose ≥6.1 mmol/L or 2-h post glycemic load blood glucose ≥7.8 mmol/L and/or those who have been diagnosed with diabetes mellitus and treated for the disease; (3) Hypertension: blood pressure ≥130/85 mmHg (1 mmHg = 0.133 kPa) and/or those who have been identified and treated for hypertension; (4) fasting triglycerides (TG) ≥1.70 mmol/L; (5) fasting high-density lipoprotein cholesterol (HDL-C) <l.04 mmol/L.

Compared with internationally accepted diagnostic criteria (e.g., the International Diabetes Federation [IDF] 2006 criteria (8) and the National Cholesterol Education Program Adult Treatment Panel III [ATP III] criteria (9)), the 2024 Criteria of the Chinese Diabetes Society (CDS) differ primarily in two dimensions: waist circumference thresholds and glycemic diagnostic criteria. In terms of waist circumference thresholds, tailored to the body fat distribution characteristics of the Chinese population, the CDS 2024 Criteria set the cutoff values for central obesity at ≥90 cm in men and ≥85 cm in women, which are significantly lower than those of the IDF 2006 Criteria (≥94 cm in men and ≥80 cm in women for European populations) and the ATP III Criteria (≥102 cm in men and ≥88 cm in women for American populations).Regarding glycemic diagnostic criteria, the thresholds for fasting blood glucose and 2-hour postprandial blood glucose specified in the CDS 2024 Criteria differ notably from the IDF 2006 Criteria, which define the fasting blood glucose cutoff as ≥5.6 mmol/L.

The rationale for adopting the CDS 2024 Criteria in this study is twofold. First, formulated based on epidemiological survey data of the Chinese population, the criteria set waist circumference, glycemic, and other diagnostic thresholds that are more consistent with the obesity characteristics and metabolic disorder prevalence of the Chinese population, thereby effectively avoiding misdiagnosis or underdiagnosis caused by the direct application of criteria developed for European and American populations. Second, widely used in domestic studies on metabolic syndrome (MetS), these criteria facilitate horizontal comparison of the findings of this study with those of other relevant domestic research. They also provide a reference basis for subsequent cross-regional studies on the pathogenesis of MetS among Chinese and foreign populations.

### 2.4 Definitions

According to the People’s Republic of China Health Industry Standard for Adult Weight Determination (Standard No. WS/T 428-2013) (10), BMI <18.5 is considered to be underweight, 18.5 ≤ BMI <24.0 is considered to be normal, 24.0 ≤ BMI <28.0 is considered to be overweight, and BMI ≥28.0 is considered to be obese. Harmful drinking (11) refers to drinking behaviors in the past 12 months with an average daily alcohol intake of 61g and above for male drinkers; and 41g and above for female drinkers. Inadequate intake of vegetables and fruits (12) refers to the average daily intake of vegetables and fruits less than 400 g. Sleep deprivation (13) refers to an average daily sleep duration of less than 7 hours.

### 2.5 Statistical methods

SPSS 25.0 and R 4.4.2 were used for statistical analysis. The independent variables were statistically described using n (%) if they were count data, and the correlation between categorical independent variables and metabolic syndrome was analyzed using the χ2 test; the measured data were statistically described using x¯ ± s if they conformed to a normal distribution, and if they did not conform to a normal distribution, they were described using M (P25, P75), and correlation analyses were conducted using Pearson correlation. All survey respondents were randomly divided into training set (70%) and validation set (30%) by R language Sample function, and correlation test and multivariate logistic regression analysis were performed on the training set, to screen out the statistically significant influencing factors, and to draw the visualized column line graph prediction model. The discriminative ability of the model and the accuracy of the prediction probability were assessed by ROC curves and calibration curves. All statistical tests were performed with *P* < 0.05, as the difference was statistically significant.

## 3 Results

### 3.1 Risk factors associated with metabolic syndrome

This study included a total of 3,541 respondents, including 2,479 in the training set and 1,062 in the validation set. The prevalence of metabolic syndrome in this study population was 18.4%. All patients in the training set were divided into six groups according to age, of which 70-79 years old had the least number of patients (6.9%); Han Chinese had the highest number of patients (96.3%), 47.6% had higher education; 73% were married/cohabiting; 52.5% were overweight; 20.9% smoked, 10.3% drank alcohol; and 39.2% had insufficient fruit and vegetable intake; The proportion of people who did not get enough sleep was 24.8%; the median intake of salt was 6; and the median intake of cooking oil was 20g (**Table 1** for other information).

**Table 1:** Results of correlation test between metabolic syndrome and its risk factors.

Features	Total(n=2479)	Non-metabolic syndrome (n=2023)	Metabolic Syndrome(n=456)	χ²/t	*P-value*
Age				126.192	<0.001
18~29	400(16.1%)	390(97.5%)	10(2.5%)		
30~39	565(22.8%)	485(85.8%)	80(14.2%)		
40~49	433(17.5%)	353(81.5%)	80(18.5%)		
50~59	491(19.8%)	375(76.4%)	116(23.6%)		
60~69	420(16.9%)	302(71.9%)	118(28.1%)		
70~79	170(6.9%)	118(69.4%)	52(30.6%)		
Gender				47.353	<0.001
Male	1205(48.6%)	917(76.1%)	288(23.9%)		
Female	1274(51.4%)	1106(86.8%)	168(13.2%)		
Ethnic				2.132	0.546
Han	2388(96.3%)	1948(81.6%)	440(18.4%)		
Man	41(1.7%)	34(82.9%)	7(17.1%)		
Hui	28(1.1%)	21(75.0%)	7(25.0%)		
Other ethnic	22(0.9%)	20(90.9%)	2(9.1%)		
Educational level				29.123	<0.001
No education	17(0.7%)	11(64.7%)	6(35.3%)		
Did not complete elementary school	31(1.3%)	22(71.0%)	9(29.0%)		
Elementary schools	69(2.8%)	51(73.9%)	18(26.1%)		
Junior high school	414(16.7%)	312(75.4%)	102(24.6%)		
High school/middle school/technical school	695(28.0%)	564(81.2%)	131(18.8%)		
Junior college	557(22.5%)	465(83.5%)	92(16.5%)		
Undergraduate	642(25.9%)	551(85.8%)	91(14.2%)		
Postgraduate and above	54(2.2%)	47(87.0%)	7(13.0%)		
Marriage				82.022	<0.001
Unmarried	526(21.2%)	500(95.1%)	26(4.9%)		
Married/cohabiting	1809(73.0%)	1415(78.2%)	394(21.8%)		
Widowhood	51(2.1%)	40(78.4%)	11(21.6%)		
Divorce/separation	93(3.8%)	68(73.1%)	25(26.9%)		
Profession				129.479	<0.001
Agriculture, forestry, fisheries and water production	24(1.0%)	16(66.7%)	8(33.3%)		
Operation of production and transportation equipment	50(2.0%)	36(72.0%)	14(28.0%)		
Commerce, services	325(13.1%)	276(84.9%)	49(15.1%)		
State organs, party organizations, enterprises, institutions	90(3.6%)	76(84.4%)	14(15.6%)		
Clerical and related personnel	276(11.1%)	211(76.4%)	65(23.6%)		
Professional and technical personnel	337(13.6%)	303(89.9%)	34(10.1%)		
Other workers	339(13.7%)	260(76.7%)	79(23.3%)		
Student at school	350(14.1%)	342(97.7%)	8(2.3%)		
Unemployed	42(1.7%)	31(73.8%)	11(26.2%)		
Domestic work	40(1.6%)	31(77.5%)	9(22.5%)		
Retirement	606(24.4%)	441(72.8%)	165(27.2%)		
BMI grouping				298.679	<0.001
Normal	1059(42.7%)	1000(94.4%)	59(5.6%)		
Overweight	860(34.7%)	644(74.9%)	216(25.1%)		
Obesity	441(17.8%)	264(59.9%)	177(40.1%)		
Underweight	119(4.8%)	115(96.6%)	4(3.4%)		
Smoking				29.665	<0.001
No	1961(79.1%)	1643(83.8%)	318(16.2%)		
Yes	518(20.9%)	380(73.4%)	138(26.6%)		
Harmful drinking				25.961	<0.001
No	2223(89.7%)	1844(83.0%)	379(17.0%)		
Yes	256(10.3%)	179(69.9%)	77(30.1%)		
Inadequate intake of fruits and vegetables				4.370	0.037
No	1034(41.7%)	866(83.8%)	168(16.2%)		
Yes	1445(58.3%)	1157(80.1%)	288(19.9%)		
Lack of sleep				10.251	0.001
No	1863(75.2%)	1547(83.0%)	316(17.0%)		
Yes	616(24.8%)	476(77.3%)	140(22.7%)		
Salt intake	6(5, 6)	6(5, 6)	6(5, 6)	0.009	0.639
Oil intake	20(6, 30)	15(6, 25)	20(6, 30)	-0.013	0.510

The results of the correlation analysis between training-focused metabolic syndrome and its risk factors are shown in **Table 1**. A total of 13 risk factors were included in this study, and the analysis found that the older the residents were, the higher the prevalence of metabolic syndrome was (*P* < 0.05); the prevalence of metabolic syndrome was higher in men than in women (23.9% vs. 13.2%, *P* < 0.05); the prevalence of metabolic syndrome decreased with the higher education level (*P* < 0.05); the prevalence of metabolic syndrome among people with different marital status was different (*P* < 0.05), and the prevalence of unmarried people (4.9%) was lower than that of other people; the prevalence of metabolic syndrome among people with different occupations differed (*P* < 0.05), and those who were engaged in agriculture, forestry, animal husbandry, fishery and water conservancy production accounted for the highest percentage (33.3%); the prevalence of metabolic syndrome increased with the increase of BMI (*P* < 0.05), and the prevalence of it was as high as 40.% among obese patients; the prevalence of current The prevalence of metabolic syndrome was higher in current smokers than in non-smokers (26.6% vs 16.2%, *P* < 0.05); the prevalence of hazardous drinking was higher than non-hazardous drinking (30.1% vs 17.0%, *P* < 0.05); the prevalence of metabolic syndrome was higher in people with insufficient intake of vegetables and fruits than in people with adequate intake of vegetables and fruits (19.9% vs 16.5%, *P* < 0.05); the prevalence of metabolic syndrome was higher in people with an average daily The prevalence of metabolic syndrome was higher in those with less than 7 hours of sleep per day than in those with more than or equal to 7 hours of sleep per day (22.7% vs. 17.0%, *P* < 0.05). There were no significant differences in ethnicity, salt intake, and oil intake between patients with and without metabolic syndrome (*P*>0.05).

### 3.2 Results of multivariate logistic regression analysis

Logistic regression models were constructed using Forward Selection (FSS) with the statistically significant variables in the correlation analysis as independent variables and the presence of metabolic syndrome as the dependent variable. The results of the covariance diagnosis showed that the tolerance of each variable was >0.1, the variance inflation factor was <3, and there was no covariance between the respective variables (**Table 2**). The results of the multivariate logistic regression analysis showed that age, gender, marital status, occupation, BMI, and alcohol consumption were independent predictors of metabolic syndrome (*P* < 0.05). The older the age, the higher the risk of metabolic syndrome (OR>1 for 18-29 years old as reference), and the prevalence risk of people aged 70-79 years was 5.601 times higher than that of people aged 18-29 years old; the prevalence risk of men was 1.827 times higher than that of women; and the prevalence risk of metabolic syndrome was significantly higher than that of the normal population for those who were overweight (OR = 4.865) and obese (OR = 11.482); The prevalence rate of harmful alcohol consumption was 1.673 times higher than that of the normal population; the prevalence risk of the widowed, married/cohabiting, and divorced/separated populations was 1.311, 2.146, and 3.034 times higher than that of the unmarried population, respectively; and the prevalence rate of the agriculture, forestry, animal husbandry, fisheries, and water conservancy production occupational groups, and the unemployed population was higher than that of the other occupational groups (for details, see **Table 3**).

**Table 2:** Covariance diagnosis of independent variables.

Variation	Tolerances	Variance inflation factor(VIF)
Age	0.697	1.436
Gender	0.919	1.088
Marriage	0.793	1.261
Profession	0.827	1.209
BMI	0.999	1.001
Harmful drinking	0.929	1.077

**Table 3:** Multivariate logistic regression analysis of risk factors for metabolic syndrome.

Features	*β*	OR	95%CI	*P-value*
Age				
18~29				0.010
30~39	0.640	1.897	0.420-8.575	
40~49	0.831	2.295	0.497-10.602	
50~59	1.181	3.258	0.706-15.040	
60~69	1.455	4.284	0.900-20.388	
70~79	1.723	5.601	1.139-27.538	
Gender				
Female				0.000
Male	0.603	1.827	1.391-2.430	
Educational level				
No education				0.570
Did not complete elementary school	-0.886	0.412	0.094-1.804	
Elementary schools	-0.966	0.381	0.105-1.377	
Junior high school	-1.104	0.331	0.102-1.076	
High school/middle school/technical school	-0.995	0.370	0.114-1.200	
Junior college	-0.874	0.417	0.127-1.368	
Undergraduate	-1.150	0.317	0.096-1.049	
Postgraduate and above	-0.987	0.373	0.085-1.627	
Marriage				
Unmarried				0.026
Married/cohabiting	0.764	2.146	1.155-3.990	
Widowhood	0.271	1.311	0.482-3.567	
Divorce/separation	1.110	3.034	1.360-6.766	
Profession				
Agriculture, forestry, fisheries and water production				0.002
Operation of production and transportation equipment	-0.323	0.724	0.214-2.446	
Commerce, services	-0.956	0.384	0.135-1.095	
State organs, party organizations, enterprises, institutions	-0.852	0.426	0.130-1.399	
Clerical and related personnel	-0.123	0.885	0.308-2.538	
Professional and technical personnel	-1.045	0.352	0.119-1.036	
Other workers	-0.270	0.763	0.272-2.143	
Student at school	-0.893	0.410	0.066-2.543	
Unemployed	-0.041	0.960	0.275-3.345	
Domestic work	-0.363	0.696	0.187-2.581	
Retirement	-0.401	0.670	0.233-1.927	
BMI grouping				
Normal				0.000
Overweight	1.582	4.865	3.537-6.692	
Obesity	2.441	11.482	8.123-16.228	
Underweight	0.126	1.135	0.388-3.315	
Smoking				
No				0.246
Yes	0.180	1.197	0.883-1.623	
Harmful drinking				
No				0.004
Yes	0.514	1.673	1.177-2.376	
Inadequate intake of fruits and vegetables				
No				0.204
Yes	0.159	1.172	0.918-1.497	
Lack of sleep				
No				0.448
Yes	0.100	1.105	0.853-1.432	

### 3.3 Column chart construction

**Figure 1:**
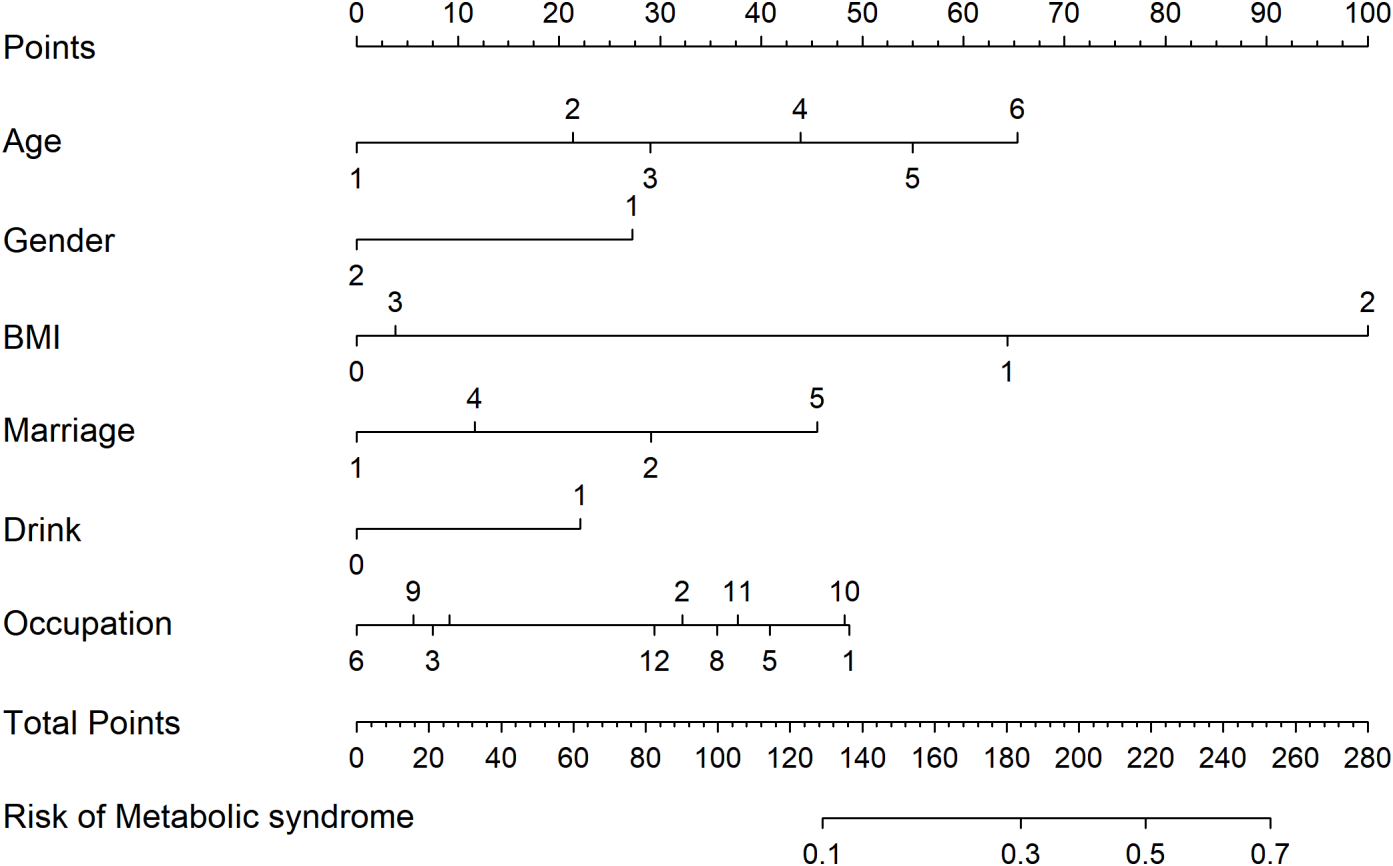
Column line graph for predicting risk of metabolic syndrome. Description of assignment: Age: 1 = 18~29 years old, 2 = 30~39 years old, 3 = 40~49 years old, 4 = 50~59 years old, 5 = 60~69 years old, 6 = 70~79 years old; Sex: Male=1, Female-2; BMI: 0=Normal, 1=Overweight, 2=Obesity, 3=Underweight; Marriage: 1=Unmarried, 2=Married/Cohabiting, 4= Widowhood, 5=Divorced/Separated; Harmful drinking: 0=No, 1=Yes; Occupation: 1=Agriculture, forestry, animal husbandry, fishery and water conservancy production, 2=Production, transportation equipment operation, 3=Commercial, service industry, 4=State organs, party organizations, enterprises, institutions, 5=Clerical and related personnel, 6=Professionals and technicians, 8=Other laborers, 9=Students in school, 10=Not in the workforce, 11=Household work, 12=Retired.

Based on the results of logistic regression analysis, six factors (age, gender, marital status, occupation, BMI, and alcohol consumption) that have an impact on the prevalence of metabolic syndrome were selected to construct a column chart to predict the risk of metabolic syndrome prevalence. **Figure 1** illustrates that BMI and age have the most significant influence on the development of metabolic syndrome. The age group of 18-29 years old, women, people with normal BMI, unmarried people, non-drinking people, and professional and technical people are the protective factors for metabolic syndrome, and the rest are risk factors.

### 3.4 Validation of the column line plots

**Figure 2:**
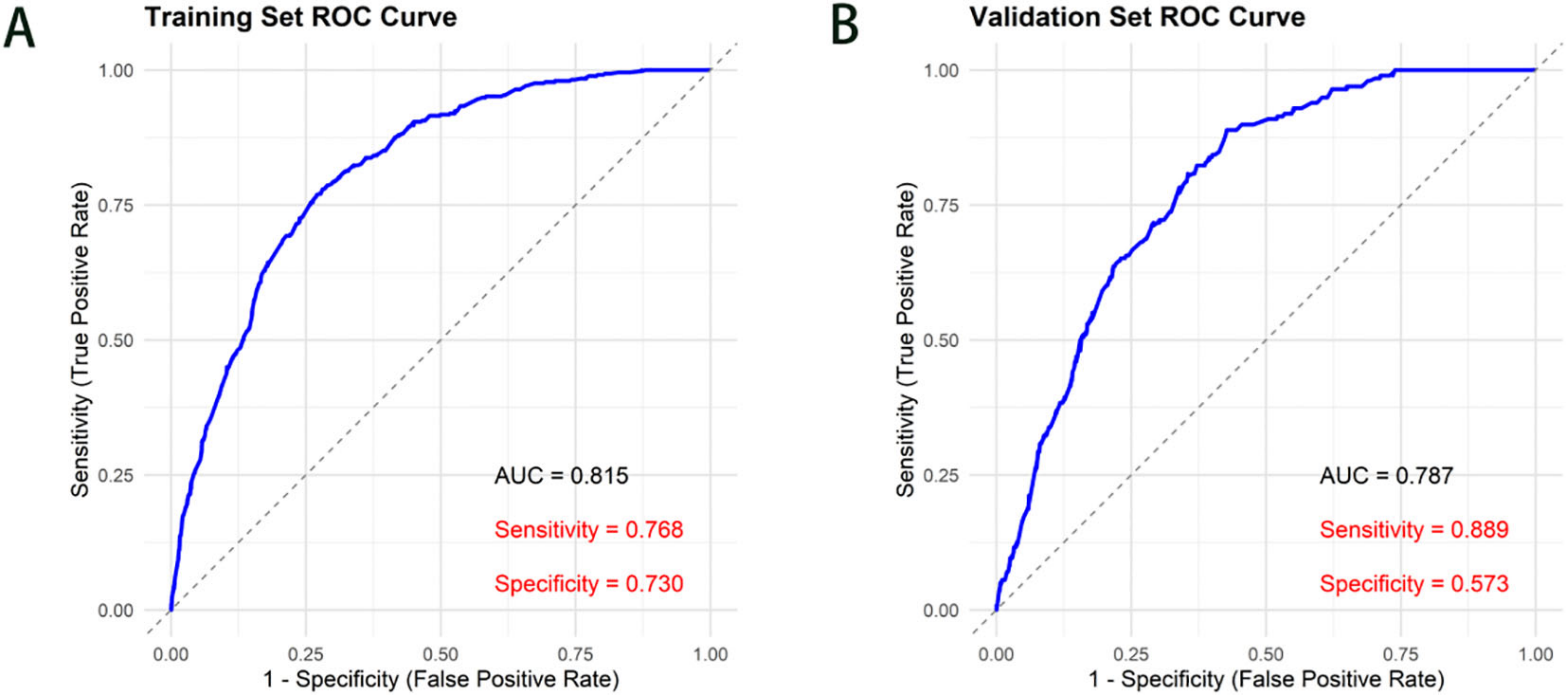
Validation of ROC curves for metabolic syndrome line graphs. **(A)** Training Set ROC Curve. **(B)** Validation Set ROC Curve; AUC, area under the curve.

The ROC curves and calibration curves were used to validate the column line plots. **Figure 2** shows the results of ROC curve analysis: area under the curve of subjects’ work characteristics for the training set ROC curve AUC = 0.815 (95% CI 0.795-0.836), and for the validation set ROC curve AUC = 0.787 (95% CI 0.756-0.818), which indicates that the model predicts good performance with high discriminatory power; the sensitivity of the training set is 0.768 and the specificity is 0.730; the sensitivity of the validation set is 0.889 and the specificity is 0.573.

**Figure 3:**
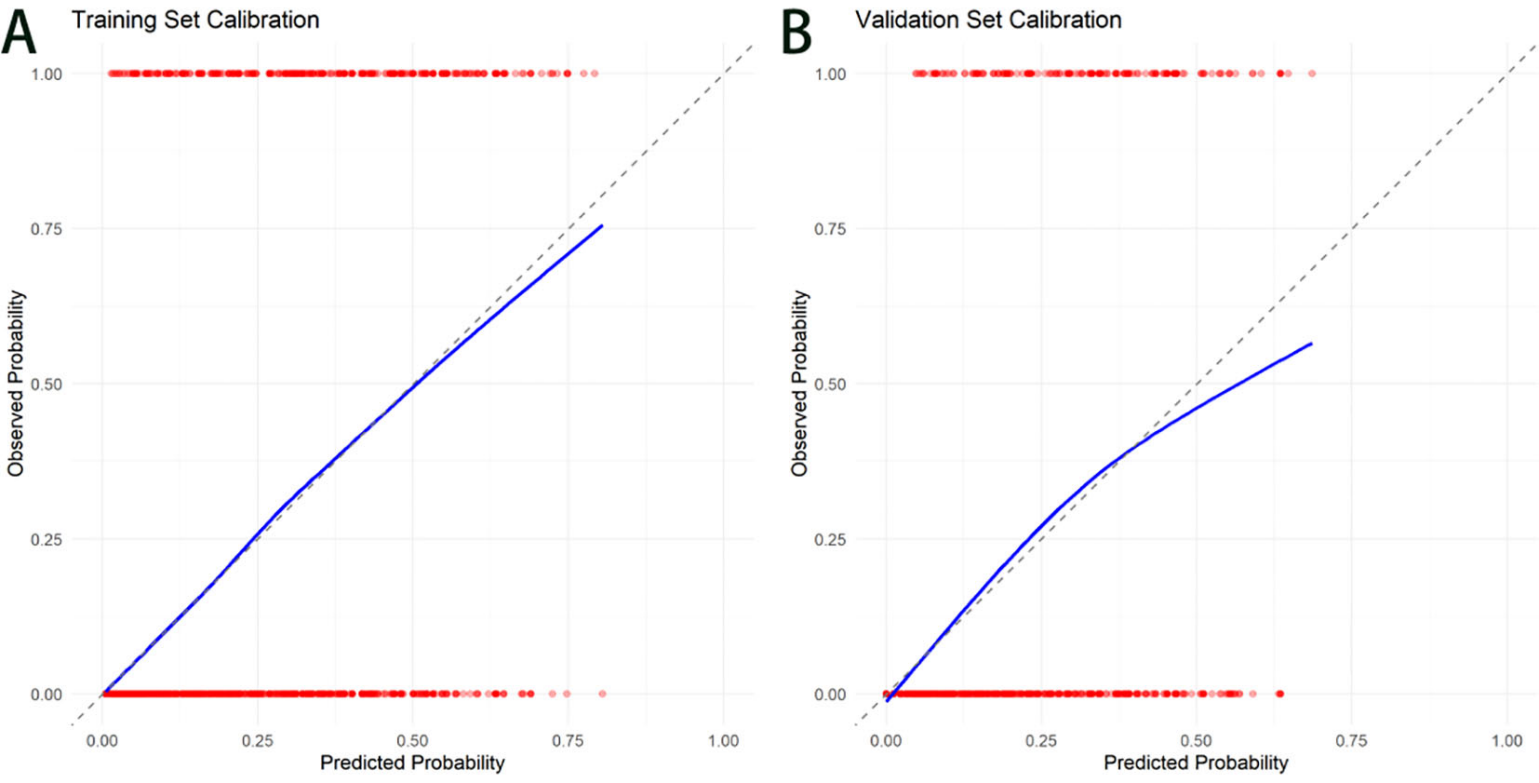
Calibration curves for the metabolic syndrome line graphs. **(A)** Training Set Calibration. **(B)** Validation Set Calibration.

**Figure 3** illustrates the results of the calibration curve analysis, demonstrating good calibration performance in both the training and validation sets. For the training set, the calibration slope derived via the Bootstrap method (with 1000 resamples) was 1.002 (95% CI: 0.987–1.017), and the calibration intercept was 0.005 (95% CI:0.008–0.018). These results indicated an excellent fitting performance of the model for the training data, which was further supported by a non-significant Hosmer-Lemeshow test result (p = 0.127). The Brier score and Mean absolute error (MAE) of the training set were 0.120 and 0.240, respectively. In the validation set, the initial model exhibited slight overfitting, as evidenced by a calibration slope of 0.761 and a calibration intercept of −0.242. After probability calibration using Platt Scaling, the calibration metrics of the validation set were notably improved: the calibration slope was adjusted to 0.92 (95% CI: 0.88–0.96), and the calibration intercept was −0.03 (95% CI: −0.07–0.01). With a Hosmer-Lemeshow test p-value of 0.082, the calibrated model demonstrated favorable calibration performance. The Brier score and MAE of the validation set were 0.136 and 0.256, respectively.

## 4 Discussion

The prevalence of metabolic syndrome among 18-79 year olds in this study was 18.4%, which is similar to the International Diabetes Federation’s statistic that about 20%-25% of adults worldwide have metabolic syndrome (14). As a significant risk factor for the prevalence of cardiovascular disease, metabolic syndrome has become one of the most important public health problems in the world, and its prevalence is increasing year by year. The results of a cross-sectional study in China, which included 158,274 study participants aged 18 years or older, showed that the prevalence of metabolic syndrome increased from 15.5% in 2012 to 20.0% in 2021 (15), so understanding the risk factors of metabolic syndrome, to recognize and take intervention measures at an early stage, is the key to preventing and controlling the development of its occurrence.

Metabolic syndrome is the result of a combination of genetic, metabolic, and socio-behavioral factors, and this study confirms that overweight, obesity, older age, male sex, harmful alcohol consumption, some marital status, and specific occupation are independent risk factors for metabolic syndrome. Among them, age and gender are uncontrollable factors. The column-line graph prediction model reflects the significant influence of age on metabolic syndrome, the older the age, the higher the risk of metabolic syndrome. The risk of metabolic syndrome for people aged 60-79 years is 4.284-5.601 times higher than that of people aged 18-29 years. Several studies have shown that age is also associated with metabolic syndrome (16–18), and that increased body fat, especially visceral obesity, in older adults may exacerbate insulin resistance, in addition to the decline in mitochondrial function that accompanies ageing. Therefore, the increasing prevalence of metabolic syndrome worldwide may be related to the aging of the population.

The risk of prevalence was higher in males than females in the present study (OR = 1.827), which is supported by many other studies (2, 19), but the prevalence of metabolic syndrome was higher in females than in males in other studies (2, 20) and was more pronounced in the postmenopausal female population. The present study only showed that the prevalence of each gender increased with age; however, the prevalence of males was consistently higher than that of females in the same age group, which may be related to the higher concentration of behavioral risk factors in males. According to the results of the Chinese Adult Tobacco Survey 2024 (21), the prevalence of smoking in Chinese men (43.9%) was much higher than that in women (1.8%). The prevalence of smoking in men (40.2%) was similarly higher than that in women (2.7%) in the present study. Studies have shown (22) that smoking and nicotine exposure may induce a pro-inflammatory metabolic state that reduces insulin sensitivity and β-cell function. The association between smoking and metabolic syndrome was not demonstrated in this study, which may be because the association between smoking and metabolic syndrome may be affected by confounding factors such as alcohol consumption and diet, or it may be a statistical null result due to insufficient sample size, which requires verification by larger-sample studies. The prevalence of metabolic syndrome is also higher in men than in women, and the present study showed that the prevalence of metabolic syndrome was 1.673 times higher in hazardous drinkers than in non-hazardous drinkers, and that alcohol-induced increases in secretion of very-low-density-lipoproteins (VLDL), impaired lipolysis, and an increased flux of free fatty acids from the adipose tissue to the liver can lead to hypertriglyceridemia (23), which can increase the risk of developing the metabolic syndrome. The issue of gender-specific differences in the prevalence of metabolic syndrome has been controversial, and more epidemiologic and even experimental studies are needed to confirm this theory.

The column-line graph prediction model revealed that BMI was the most influential factor in metabolic syndrome, with overweight (OR = 4.865) and obesity (OR = 11.482) having the most significant effects. The greater the weight, the higher the risk of developing metabolic syndrome. Insulin resistance and central obesity have now been recognized as important factors in metabolic syndrome (24), which is consistent with the study of the pathophysiological mechanisms of metabolic syndrome: firstly, obesity-induced accumulation of adipose tissue increases the release of free fatty acids, which inhibits the insulin signaling pathway; secondly, secretion of inflammatory factors by visceral adipose (e.g., TNF-α, IL-6) induces chronic low-grade inflammation, which inhibits insulin receptor activity or block signaling (25).

Marriage and occupation do not directly influence the factors associated with metabolic syndrome. The prevalence of metabolic syndrome in this study was higher in those who have or have had a partner with a common life experience than in those who are unmarried, and the married/cohabiting population has an increased number of calorie diets and decreased exercise after marriage, leading to obesity and metabolic abnormalities; whereas, widowed and divorced populations produce inflammatory cytokines due to the stress of psychological stress, and the likes of IL-6, and TNF-α can induce insulin resistance through the blockage of the insulin signaling pathway thus inducing insulin resistance (26, 27). Agricultural, forestry, fishery, and water conservancy production, and the unemployed are high-risk groups for metabolic syndrome. Agricultural, forestry, fishery, and water conservancy producers often experience irregular working hours, insufficient sleep, and circadian rhythm disorders, which have been linked to metabolic disorders, such as impaired insulin function (28, 29). Physical inactivity among the unemployed leads to the accumulation of visceral fat and decreased insulin sensitivity, which increases the risk of metabolic syndrome.

## 5 Innovations and limitations

### 5.1 Innovations

A total of 3,541 participants were enrolled in this study via questionnaire surveys, physical examinations, and laboratory tests, with 13 risk factors evaluated. A nomogram risk prediction model was constructed based on logistic regression, and several independent risk factors were identified. The area under the receiver operating characteristic curve (AUC) was 0.815, indicating high predictive accuracy of the model. Although the prediction error (MAE = 0.240) was slightly higher than the ideal threshold, this was consistent with the multifactorial pathogenic characteristics of metabolic syndrome. A Brier score of 0.120 suggested that the model could effectively distinguish between individuals at high and low risk.

### 5.2 Limitations

First, it is a retrospective study in a localized area, and the results may be influenced by factors specific to the area (e.g., environmental, socioeconomic, and cultural practices). Meanwhile, this study can only reveal the correlation between risk factors and metabolic syndrome, but cannot confirm the causal relationship between them, which requires further verification through prospective cohort studies.

Second, the study sample was solely recruited from one district in Beijing, with a lack of data from rural areas and populations in other provinces, which largely limits the external validity of the established model. In addition, the glycated hemoglobin (HbA1c) index was not included in the study, which may affect the accuracy of the analysis results of blood glucose-related indicators.

In the future, large-sample, multicenter, multivariate (e.g., biomarkers such as inflammatory factors) prospective studies to analyze the influencing factors of metabolic syndrome will yield clearer findings and improve prediction accuracy. Additionally, multicenter and cross-regional studies are recommended, which should incorporate samples from populations of different geographic regions and both urban and rural areas to verify the applicability of this model in a broader population.

## 6 Conclusion

This study showed that the prevalence of MetS in 18–79-year-olds was 18.4%, consistent with the global average. It confirmed that overweight, obesity, advanced age, male sex, harmful alcohol consumption, specific marital statuses and occupations are independent risk factors for MetS, with BMI being the most impactful. The constructed nomogram can effectively distinguish MetS high- and low-risk groups. Given most risk factors are modifiable, high-risk groups and those associated with indirectly controllable factors can reduce MetS risk via interventions like lifestyle adjustments and regular work-rest schedules. This study provides important reference for formulating targeted public health intervention strategies and positive guidance for MetS prevention and control.

## Data Availability

The raw data supporting the conclusions of this article will be made available by the authors, without undue reservation.
